# Differences in the predictors of the resilience between carers of people with young- and late-onset dementia: a comparative study

**DOI:** 10.1590/1980-5764-DN-2021-0093

**Published:** 2022-05-23

**Authors:** Nathália Ramos Santos Kimura, Maria Alice Tourinho Baptista, Marcia Cristina Nascimento Dourado

**Affiliations:** 1 Universidade Federal do Rio de Janeiro, Instituto de Psiquiatria, Centro de Doença de Alzheimer e Distúrbios Relacionados, Rio de Janeiro RJ, Brazil.

**Keywords:** Caregivers, Resilience, Psychological, Dementia, Cuidadores, Resiliência Psicológica, Demência

## Abstract

Resilience is a subjective process related to both protective and risk factors, external and internal to the individual. Considering the psychosocial differences between young-onset dementia (YOD) and late-onset dementia (LOD) groups, carers’ resilience may not be understood in the same way in both groups.

**Objective:**

The aim of this study was to compare the resilience of carers in YOD and LOD and to examine which factors might be associated with resilience in both groups of carers.

**Methods:**

The study was conducted with 120 people with dementia (49 YOD) and their primary carers. The carers had their resilience, quality of life, depressive symptoms, and burden assessed and answered the sociodemographic questionnaire. We assessed care recipients’ global cognition, dementia severity, social cognition, facial expression recognition, awareness of disease, the ability to perform activities of daily living, depressive symptoms, and neuropsychiatric symptoms. For data analysis, unpaired two-tailed Student’s *t*-test and linear regressions were conducted.

**Results:**

Resilience did not differ between groups (p=0.865). Resilience was inversely related to carers’ depressive symptoms in both YOD (p=0.028) and LOD (p=0.005) groups. The carers’ schooling (p=0.005), duration of disease (p=0.019), and depressive symptoms of care recipient (p<0.001) were related to carers’ resilience only in LOD group.

**Conclusions:**

The context of care, clinical status of the care recipient, and mental health resources affected the carers’ resilience in the LOD group. Conversely, resilience seems to be affected only by carers’ mental health in the YOD group. The understanding of these differences is crucial for the developing of intervention strategies.

## RESUMO

A resiliência é um processo subjetivo relacionado a fatores de proteção e risco, externos e internos ao indivíduo. Considerando as diferenças psicossociais entre demência de início precoce (DIP) e demência de início tardio (DIT), a resiliência dos cuidadores pode não ser entendida da mesma maneira em ambos os grupos.

**Objetivo:**

O objetivo deste estudo é comparar a resiliência de cuidadores de DIP e DIT e examinar quais fatores poderiam estar associados à resiliência em ambos os grupos de cuidadores.

**Métodos:**

O estudo foi realizado com 120 pacientes com demência (49 DIP) e seus cuidadores primários. Os cuidadores tiveram sua resiliência, qualidade de vida, sintomas depressivos e sobrecarga avaliados e responderam ao questionário sociodemográfico. Avaliou-se a cognição global, a severidade da demência, a cognição social, o reconhecimento da expressão facial, a consciência da doença, a funcionalidade em atividades de vida diária, e os sintomas depressivos e neuropsiquiátricos dos pacientes. Para a análise dos dados, foram realizados teste *t* de Student bicaudal não pareado e regressões lineares.

**Resultados:**

Não houve diferença na resiliência entre os grupos (p=0,865). A resiliência foi inversamente relacionada com sintomas depressivos dos cuidadores em DIP (p=0,028) e DIT (p=0,005). A escolaridade do cuidador (p=0,005), tempo de doença (p=0,019) e sintomas depressivos dos pacientes (p<0,001) foram relacionados à resiliência apenas no grupo DIT.

**Conclusões:**

O contexto do cuidado, o estado clínico do paciente e os recursos de saúde mental afetaram a resiliência do cuidador no grupo DIT. Em contrapartida, a resiliência parece ser afetada apenas pela saúde mental do cuidador em DIP. O entendimento dessas diferenças é crucial para o desenvolvimento de estratégias de intervenção.

## INTRODUCTION

Across the world, care for people with dementia is frequently given by family members^1^. Carers of people with dementia are often understood as the invisible second patients^2^. The negative physical and mental health consequences of caring for a person with dementia have been well documented^2–4^.

Carers of people with dementia are a group that requires attention due to high levels of stress, distress, and burden^5,6^. In addition, some studies indicate that these carers contemplate suicide at more than four times the rate of the general population (with some even contemplating homicide suicide)^7–9^ and that they have an increased risk of mortality^10^.

Nonetheless, there are differences within the carers’ group. The most of cases of dementia occur among older adults, although people under 65 years may also develop the dementia^11^. Previous studies reported that carers of people with young-onset dementia (YOD) present more severe depressive symptoms and burden than carers of people with late-onset dementia (LOD)^12–16^. Usually, carers of people with YOD are unready for the carer’s tasks and experience increased burden when compared to carers of people with LOD^12–16^.

Despite this, some carers, even suffering great caregiving demands, seem to cope fairly well and present fewer negative outcomes of caregiving than others^17^. Positive aspects of caregiving are reported in some studies, including an improved rapport between carer and people with dementia and the carer’s feeling of accomplishment^18^. This aspect may potentially be understood as an indicator of resilience.

Resilience is described as the process of well adjustment in cases of trauma, adversity, threats, tragedy, or even a considerable cause of stress^19^. Some studies consider the experience of caregiving as adversity^20,21^, while other studies consider the negative consequences of caregiving reported by carers of people with dementia as the adversity that carers must adjust to or overcome^21,22^.

The resilience may be considered a dynamic process^23^ involving both protective and risk factors, external and internal to the individual^24^. Resilience involves the interaction of protective factors such as confidence in caregiving, problem-solving skills, a strong sense of religion or spirituality, and social support^25,26^. The predominance of protective factors may make the carer more resilient. More resilient carers generally cope better with the changes in people with dementia behavior because they seem to be better prepared for the inexorable changes arisen from the dementia process.

Existing research suggests that resilience is inversely associated with burden, anxiety, and depression and is viewed as an essential factor in suicide prevention^5,22,27–30^. Resilience has been found to be positively related to factors that promote positive outcomes, including self-efficacy, self-esteem, problem-focused coping, mastery, flexibility, and adaptation^5,22,27^. In addition, resilience provides an optimal psychological adaptation and improves other coping strategies in feedback to the demands of dementia care^31^.

In the current literature, there is not a study that compares the resilience of carers of people with YOD with the resilience of carers of people with LOD. Taking that resilience is a subjective process and the psychosocial diversity among both groups of carers, this study hypothesizes that resilience is poorer in the YOD group than in the LOD group. Therefore, the aim of this study was to compare the resilience of carers in YOD and LOD and to examine which factors might be associated with resilience in both groups of carers.

## METHODS

This observational cross-sectional descriptive study was performed between February 2016 and December 2019 in the Center for Alzheimer’s Disease outpatient clinic of Institute of Psychiatry of Federal University of Rio de Janeiro, Brazil.

### Participants

The sample consisted of 120 dyads of home-dwelling outpatients with dementia and their primary carers, with 49 in the YOD group and 71 in the LOD group.

Dementia was diagnosed by a psychiatrist according to the criteria established by the *Diagnostic and Statistical Manual of Mental Disorders, Fifth Edition* (DSM-5)^32^. In this study, 114 people were diagnosed with Alzheimer’s disease and 6 people were diagnosed with vascular dementia.

People with mild-to-severe dementia according to Clinical Dementia Rating (CDR)^33^ and those who score below 26 in Mini-Mental State Examination (MMSE) were included in this study^34^. The exclusion criteria were people with severe communication problems, traumatic events, and alcohol or substance dependency or abuse.

The primary carer was the principal person for the care of people with dementia, and they should be able to give elaborate information about the care recipients and be an informal carer. Carers with a reported history of cognitive or psychiatric disorders prior to the dementia diagnosis were not included in this study.

This study was authorized by the ethics committee of the Institute of Psychiatry of the Federal University of Rio de Janeiro. At the outpatient clinic, all people with dementia and their carers signed informed consent forms before the assessment, according to the Declaration of Helsinki.

### Procedure

Each person with dementia completed assessments of global cognition, social cognition, facial expression recognition, and awareness of disease. Additional data, including the ability to perform activities of daily living, depressive symptoms, neuropsychiatric symptoms, dementia severity, and sociodemographic data, were obtained through questionnaires and instruments answered by the carer. The carers also had their resilience, quality of life (QoL), depressive symptoms, and burden evaluated and answered the sociodemographic questionnaire.

### Measures

#### Cognition

Mini-Mental State Examination (MMSE): This instrument is composed of 30 items that measures orientation, comprehension, learning, short-term memory, language, and basic motor skills. The total score ranges from 0 to 30, with lower scores signaling more impaired cognition^34^.

#### Severity of dementia

Clinical Dementia Rating (CDR): This test assesses the severity of dementia. The stage ranges from 0 (no dementia) to 3 (severe dementia) according to the degree of cognitive, activities of daily livings, and behavioral impairment^33^.

#### Functionality

The Pfeffer Functional Activities Questionnaire (PFAQ): This inventory evaluates the activities of daily living. The score for each item ranges from normal (0) to dependent (3), with a total of 30 points. Higher score suggests greater functional impairment^35^.

#### Depressive symptoms

The Cornell Scale for Depression in Dementia (CSDD): This scale assesses circadian functions, physical signs, mood, and behavioral symptoms related to depressive symptoms between people with dementia. The total rating ranges from 0 to 38. Score>13 suggests the presence of depressive symptoms^36^.

#### Neuropsychiatric symptoms

Neuropsychiatric Inventory (NPI): This inventory assesses delusions, hallucinations, agitation, apathy, anxiety, depression, euphoria, irritability, disinhibition, aberrant motor behavior, change in appetite, and nighttime behavior disturbances. Each item is assessed in relation to their frequency (1=absent to 4=frequently) and intensity (1=mild to 3=severe). The total rating ranges from 0 to 144. Higher score suggests greater levels of neuropsychiatric symptoms. We used 12 items^37^.

#### Awareness of disease

Assessment Scale of Psychosocial Impact of the Diagnosis of Dementia (ASPIDD): The ASPIDD is a 30-question scale centered on people with dementia and carer reports. This scale was designed to assess awareness of disease based on the scoring of discrepant responses through four domains, namely, cognitive functioning, health condition, emotional state, social functioning/relationships, and instrumental and basic activities of daily living. The carer responds the same questions as the people with dementia, with one point being scored for each discrepant response. The ratings of awareness range from preserved (0–4), mildly impaired (5–11), moderately impaired (12–17), to absent (<18)^38^.

#### Social and emotional functioning

Social and Emotional Questionnaire: This instrument is composed of 30 items based on 5 factors: recognition of emotion, empathy, social conformity, antisocial behavior, and sociability. The ratings for each item range from strongly disagree (1) to strongly agree (5). We used the carer’s version about people with dementia emotional and social current functioning. The score is measured on five-point Likert scale, ranging from “strongly disagree” (1) to “strongly agree” (5). Lower score indicates more impaired social and emotional functioning^39^.

#### Facial expression recognition ability

Facial Expression Recognition Ability Scale (FACES): We used an adaptation of an experimental task developed by Shimokawa et al. Task 1 investigates the visuoperceptual ability to identify faces. Task 2 evaluates the ability to comprehend facial emotions. Task 3 examines whether subjects can recognize the expression of emotion conceptually. Task 4 assesses the people with dementia’s ability to comprehend the nature of a situation and the appropriate emotional state that one would experience in that situation. For each correct response, the subject receives 1 score. FACES is composed of 16 tasks, and the highest possible score is 16. Lower score suggests impaired recognition^40^.

### Carer measures

#### Resilience

Resilience Scale by Wagnild and Young: This original resilience measure, considered the “gold standard” for resilience evaluation, has 25 items that assess psychosocial adaptation to adversity. The score ranges from 25 to 175 and was classified as follows: 25–124: low; 125–145: moderate; and 146–175: high resilience^41^.

#### Quality of life

Quality of Life in Alzheimer’s Disease (QoL-AD): The QoL-AD includes 13 domains (i.e., physical health, energy, mood, living situation, memory, family, marriage, friends, you as a whole, ability to do chores, ability to do things for fun, money, and life as a whole) that are rated as poor (1), fair (2), good (3), or excellent (4). We used the carer’s QoL version (C-QoL). The total score ranges from 13 to 52. Higher score indicates better QoL^42^.

#### Burden

Zarit Burden Interview (ZBI): This assessment consists of 22 items that evaluate the impact of caring for people with dementia on the carer’s life by appointing how often the carer experiences a particular feeling: never (0), rarely (1), sometimes (2), quite frequently (3), or nearly always (4). The total score ranges from 0 to 88. Higher score indicates a higher level of burden^43^.

#### Depressive symptoms

Beck Depression Inventory (BDI): This is self-report scale, composed of 21 items based on symptoms of depression such as hopelessness and irritability, cognitions such as guilt or feelings of being punished, as well as physical symptoms such as fatigue, weight loss, and lack of interest in sex. The total score ranges from 0 to 63 and categorized as follows: 0–11: mild symptoms, 12–19: mild to moderate, 20–35: moderate to severe, and 36–63: severe symptoms^44^.

### Statistical analysis

All statistical analyses were performed with SPSS software for Windows version 23.0. The variables were inspected for normality before analysis. Initially, the descriptive analyses of all the variables were carried out by observing the means, standard deviation, and frequency (percentage) according to the type of variable studied. All analyses were performed by thematic blocks, namely, sociodemographic data of the people with dementia and carer and clinical data of people with dementia and carer. Depending on the variable of interest, we utilized t-tests for independent samples (with homoscedasticity test) and the χ^2^ test, the Fischer’s exact test, or the Mann-Whitney U test to test for significant group differences.

Multivariate linear regressions with the stepwise method were elaborated using resilience as dependent variable. All demographic and clinical variables were included as independent variables. Regression models were performed separately for YOD and LOD groups and the best models were selected according to highest explained variance of the R² and the variance inflation factor (VIF) close to 1, for the collinearity in each independent variable. For all analyses, the α level was set at p≤0.05.

## RESULTS

### Sociodemographic characteristics

The mean age of YOD people was 63.69±6.2 years. The majority of people with dementia were men (51%, n=25) and married (67.3%, n=33). While most of carers were women (83.7%, n=41). The majority of carers were wives or husbands (55.1%, n=27), with a mean age of 52.06±14.2 years.

The mean age of LOD people was 79.65±5.7 years. Most of the people with dementia were women (67.6%, n=48). The majority were widowers (42.3%, n=30). Also, most of the carers in this group were women (73.2%, n=52). Regarding the kinship, the majority were daughters or sons (54.9%, n=39), with a mean age of 57.89±14.3 years.

Table 1 lists the sociodemographic characteristics of people with dementia and carers.

**Table 1:** Sociodemographic characteristics of people with dementia and carers according to age of onset.

			YOD (n=49)	LOD (n=71)	p-value
PwD	Female, n (%)		24 (49.0)	48 (67.6)	
	Age, mean (SD)		63.69 (6.2)	79.65 (5.7)	<0.001*
	Age of onset, mean (SD)		57.73 (4.9)	75.03 (6.0)	<0.001*
	Duration of disease, mean (SD)		5.76 (3.1)	4.62 (3.3)	0.062
	Schooling, mean (SD)		10.00 (4.1)	7.15 (4.0)	<0.001*
	CDR, n (%)	Mild	20 (40.8)	47 (66.2)	
		Moderate	20 (40.8)	21 (29.6)	
		Severe	9 (18.4)	3 (4.2)	
	Marital status, n (%)	Singles	2 (4.1)	6 (8.5)	
		Married	33 (67.3)	27 (38.0)	
		Widowers	6 (12.2)	30 (42.3)	
		Divorced	8 (16.3)	8 (11.3)	
Carers	Female, n (%)		41 (83.7)	52 (73.2)	
	Age, mean (SD)		52.06 (14.2)	57.89 (14.3)	0.030*
	Schooling, mean (SD)		11.41 (3.9)	12.08 (3.2)	0.328
	Kinship, n (%)	Wives/ husbands	27 (55.1)	22 (31.0)	
		Daughters/sons	15 (30.6)	39 (54.9)	
		Others	7 (14.3)	10 (14.1)	

PwD: people with dementia; YOD: young-onset dementia; LOD: late-onset dementia; SD: Standard deviation; CDR: Clinical Dementia Rating;

* significant result.

### Clinical characteristics of dyads

Comparison between groups showed that people with YOD were more cognitively impaired according to the MMSE (p<0.001) and also had more deficits in functionality as rated on the PFAQ (p=0.046).

We did not observe a significant difference in carers’ resilience (p=0.865) and in the other clinical characteristics between both carers’ groups. Carers of both groups reported moderate to high levels of resilience. However, the YOD group of carers presented a slight level of burden and depressive symptoms than the LOD one.

The clinical characteristics of people with dementia and carers are synthesized in Table 2.

**Table 2:** Clinical characteristics of people with dementia and carers according to age of onset.

		YOD (n=49)	LOD (n=71)	p-value
PwD	MMSE (SD)	15.57 (5.8)	19.18 (5.0)	<0.001*
	ASPIDD (SD)	9.25 (5.6)	9.82 (5.4)	0.584
	SEQ C-PwD (SD)	100.90 (17.3)	105.38 (15.1)	0.146
	FACES (SD)	9.63 (4.0)	10.82 (3.0)	0.069
	CSDD (SD)	8.51 (6.0)	7.28 (5.4)	0.247
	PFAQ (SD)	20.12 (7.9)	16.96 (8.7)	0.046*
	NPI Total (SD)	21.69 (19.1)	18.85 (19.9)	0.436
Carers	QoL-AD (SD)	35.00 (5.8)	35.79 (6.7)	0.507
	ZBI (SD)	33.45 (17.4)	31.14 (16.2)	0.459
	BDI (SD)	8.43 (7.2)	7.86 (7.2)	0.674
	RS (SD)	140.67 (14.0)	140.13 (19.1)	0.865

PwD: people with dementia; YOD: young-onset dementia; LOD: late-onset dementia; SD: Standard deviation; MMSE: Mini-Mental State Examination; ASPIDD: Assessment Scale of Psychosocial Impact of the Diagnosis of Dementia; SEQ C-PwD: carers’ reports on social and emotional functioning of people with dementia; FACES: recognize facial expressions; CSDD: Cornell Scale for Depression in Dementia; PFAQ: Pfeffer Functional Activities Questionnaire; NPI: neuropsychiatric inventory; QoL-AD: Quality of life in Alzheimer’s disease scale (carers’ reports on their own quality of life); ZBI: Zarit Burden Interview; BDI: Beck Depression Inventory; RS: Resilience Scale; *significant result.

### Multivariate analyses

The linear regression model showed that lower levels of resilience of carers of people with YOD were related to higher levels of carers’ depressive symptoms (p=0.028).

The analysis of the LOD group showed that resilience was inversely related to carers’ depressive symptoms (p=0.005) and their schooling (p=0.005) and duration of disease (p=0.019). Moreover, resilience was associated with depressive symptoms of people with dementia (p<0.001). Carers reported high levels of resilience when people with dementia exhibited more depressive symptoms.

The adjusted R^2^ values and the standardized regression weights are presented in Table 3.

**Table 3:** Regression model of factors related to resilience.

	R	R^2^	Adj. R^2^	B	Beta	t	p-value
YOD	0.318	0.101	0.081				
BDI				-0.608	-0.318	-2.272	0.028
LOD	0.533	0.284	0.241				
BDI				-0.820	-0.312	-2.931	0.005
Carer’s schooling				-1.837	-0.312	-2.910	0.005
Duration of disease				-1.462	-0.256	-2.395	0.019
CSDD				0.770	0.219	2.090	0.041

B: linear coefficient; BETA: standardized beta coefficient; T: YOD: young-onset dementia; LOD: late-onset dementia; BDI: Beck Depression Inventory; SEQ PwD: Social and Emotional Questionnaire (self-reported PwD ratings); CSDD: Cornell Scale for Depression in Dementia.

## DISCUSSION

In this study, we investigated the resilience of carers of people with YOD compared to carers of people with LOD. Carers of both groups presented moderate to high levels of resilience, a fact that may clarify the lack of significant difference in the carers’ resilience between groups. We may suppose the occurrence of a positive adjustment of the carers to the conditions of care. In addition, there were no significant differences in the clinical characteristics of both carers’ groups. It is worth highlighting that they were part of a treatment center for people with dementia that provides support for their carers. The presence of an external resource seems to assist the carers in coping with the demands involved in providing care to people with dementia and to keep their levels of health.

The hypothesis of our study was not confirmed. However, our results indicate that the factors that affected resilience differ according to the age of onset of dementia.

The carers’ depressive symptoms were the only predictor of the resilience of carers of people with YOD. Also, a previous study conducted by our group found the same relationship between resilience and depressive symptoms^45^. Other studies have already shown that higher levels of resilience were related to lower levels of depressive symptoms of carers^28,46^. Therefore, in the YOD group, carers’ resilience seems not to be associated with the cognitive and clinical symptoms of the people with dementia^22^. Our findings showed that carers’ depressive symptoms were also a predictor of the resilience of carers of people with LOD. Despite the low levels of depressive symptoms of carers of both groups, the results propose that resilience may impact carers’ mental health.

Resilience may be influenced by context of care, status of the care recipient, and individual, family, and community resources^22^. Thus, our findings demonstrated the interaction between these constellation of aspects in carers’ resilience. We observed that a lower level of carers’ schooling was associated with higher resilience in the LOD group. Our study was realized in a Latin American country, which may justify this outcome. People with a lower level of schooling can be amenable to the role of carer since society demands higher levels of schooling for the formal labor market. Gaugler et al.^22^ also found a negative relationship between education and carer resilience. People with less education may be dedicated to caring tasks of their dependent family members and have more possibility to develop resilience^22^.

In the LOD group, carers’ resilience was inversely associated with the duration of disease. With the progression of the disease, the carer may develop a burden due to the increase in dependency of people with dementia. In the literature, there is a negatively strong correlation between burden and resilience^5,21,22,47,48^. Resilient carers who detected their proper ability to cope with adversity reported less burden^5^. We may hypothesize that the negative relation between resilience and duration of disease was influenced by the level of carer’s burden. Further studies should employ a path analysis approach to better understand the interface between resilience, duration of disease, and burden.

Another substantial result of our study was the effect of the depressive symptoms of people with dementia on the resilience of carers of the LOD group. Resilience enables carers to manage and respond positively to stressing caregiving conditions^22,28^. Being resilient does not mean a lack of difficulties when confronted with adversity, but that the person faces difficulties effectively^28^. Therefore, despite the presence of depressive symptoms of people with dementia, many carers may keep resilient.

The literature supports the idea that there are specific experiences and needs of carers based on the age at onset of disease of care recipient^12–16^. Our data supply insights that could enable a more significant appreciation of the resilience of carers of people with YOD and LOD and your predictors. Few studies recognize the heterogeneity of existing characteristics among carers, considering this group as a single block. The study by Ducharme et al.^49^ showed that, besides taking care of a person with dementia, carers of people with YOD are younger, which causes double stigmatization. The carers’ resilience must be understood as having particular characteristics that may vary according to YOD or LOD groups.

### Limitations

We studied a relatively small and convenience sample and this was a cross-sectional study. The inclusion of people with other dementias besides dementia due to Alzheimer’s disease was another limitation of our study. Moreover, we did not evaluate the carers’ personality traits. These factors could impact the resilience of carers in both YOD and LOD groups.

This article is the first to study about the factors related to the resilience of carers of people with YOD compared to carers of people with LOD. The context of care, the status of the care recipient, and individual resources influenced the carers’ resilience in the LOD group. Conversely, in the YOD group, carers’ resilience seems to be influenced only by individual resources.

Understanding these aspects is crucial for developing intervention strategies more appropriately designed to suit the demands of each of these groups. Furthermore, increasing the levels of carers’ resilience may mitigate the negative outcomes of caregiving, allowing caregivers to remain in the role for longer, improving the quality of care they provide, and reducing the early institutionalization of people with dementia.
